# Neuroprotection by quercetin *via* mitochondrial function adaptation in traumatic brain injury: PGC‐1α pathway as a potential mechanism

**DOI:** 10.1111/jcmm.13313

**Published:** 2017-12-04

**Authors:** Xiang Li, Handong Wang, Guodao Wen, Liwen Li, Yongyue Gao, Zong Zhuang, Mengliang Zhou, Lei Mao, Youwu Fan

**Affiliations:** ^1^ Department of Neurosurgery Jinling Hospital Medical School of Medicine Nanjing University Nanjing Jiangsu Province China

**Keywords:** quercetin, mitochondria, PGC‐1α, traumatic brain injury, neuroprotection

## Abstract

The aim of this study was to investigate the neuroprotective effects of quercetin in mouse models of traumatic brain injury (TBI) and the potential role of the PGC‐1α pathway in putative neuroprotection. Wild‐type mice were randomly assigned to four groups: the sham group, the TBI group, the TBI+vehicle group and the TBI+quercetin group. Quercetin, a dietary flavonoid used as a food supplement, significantly reduced TBI‐induced neuronal apoptosis and ameliorated mitochondrial lesions. It significantly accelerated the translocation of PGC‐1α protein from the cytoplasm to the nucleus. In addition, quercetin restored the level of cytochrome c, malondialdehyde and superoxide dismutase in mitochondria. Therefore, quercetin administration can potentially attenuate brain injury in a TBI model by increasing the activities of mitochondrial biogenesis *via* the mediation of the PGC‐1α pathway.

## Introduction

TBI is the leading cause of death and disability in young adults 1, 2, 3, 4. Primary insult mainly determines the outcome; however, evidence from an increasing number of studies has proven that secondary damage caused by pathological processes, including loss of ionic homoeostasis, excitotoxicity and inflammation, exhibits sensitivity to therapeutic interventions 5, 6, 7. Among these processes, production of reactive oxygen species (ROS) due to oxidative stress significantly affects the secondary damage 8 because of excessive production of ROS and damage to the antioxidant system. Mitochondria are identified as the main source of cellular ROS, and their impairment increases ROS production. Such impairment damages mitochondrial proteins, DNA and lipids, thereby disrupting cellular Ca^2+^ homoeostasis, inducing apoptosis, and causing metabolic failure 9, 10. The degree of mitochondrial injury or dysfunction can significantly determine cell survival; otherwise, death would spread from mitochondrion to mitochondrion, damaging the cell itself. Despite recent improvement in understanding the pathological process of TBI, effective pharmacological therapies remain limited.

Quercetin, a natural aglycone flavonoid occurring in a wide variety of fruits and vegetables, including apples, red onions and berries, has received considerable interest because of its anti‐inflammatory, anti‐proliferative and anti‐atherosclerotic effects 11, 12. Likewise, it can affect mitochondrial biogenesis by modulating enzymes and transcription factors in the inflammatory signalling cascade 12, 13. In addition, quercetin can modulate mitochondrial biogenesis by reducing ROS production in various cell types.

The peroxisome proliferator‐activated receptor (PPAR) γ coactivator‐1 (PGC‐1) family of transcriptional coactivators is emerging as a ‘molecular switches’ in many metabolic pathways. PGC‐1α, the first characterized PGC‐1 family member, plays a pivotal role in various central nervous system diseases 14. PGC‐1α facilitated mitochondrial transcription factor A or TFAM, which transcribes structural proteins as well as proteins involved in mitochondrial DNA transcription, translation and repair 15, 16, 17. The nuclear PGC‐1α protein level significantly increased in the neurons of the cerebral cortex post‐TBI 18. However, these results suggest that PGC‐1α can significantly affect the brain post‐TBI. The effectiveness of quercetin in the treatment of numerous neuronal diseases has been established. Moreover, the association between the neuroprotective effects of quercetin and the activation of the PGC‐1α pathway draws interest.

This study aimed to investigate the ameliorative effect of quercetin on TBI‐induced mitochondrial injury and to evaluate whether the neuroprotective effects of quercetin are related to the activation of the PGC‐1α pathway.

## Materials and methods

### Animals

Male ICR mice (Experiment Animal Centre of Nanjing Medical University, Jiangsu, China) aged 6–8 weeks and weighing 28–32 g were used in this study. The mice were housed throughout the study under the following conditions: 23 ± 1°C, a 12 hrs light/dark cycle, and *ad libitum* access to food and water. Experiment protocols were approved by the Animal Care and Use Committee of Nanjing University and conformed to the Guide for the Care and Use of Laboratory Animals by the National Institutes of Health.

### TBI Model

Marmarou's weight‐drop model was used in this study 19. Mice were briefly anaesthetized with intraperitoneal injections of chloral hydrate (400 mg/kg) and then placed on the platform directly under the weight‐drop device. The fascia was reflected to expose the skull after a 1.5 cm midline longitudinal scalp incision was made. After the left anterior frontal area (1.5 mm lateral to the midline on the mid‐coronal plane) was located as the impact area, a 200 g weight was released from a height of 2.5 cm onto the skull 19. The scalp wound was subsequently sutured. All of the mice were then returned to quondam cages. The mice in the sham group underwent the same procedures except for the weight drop.

### Experimental design

All the ICR mice were divided into six groups (*n* = 24 each group): sham, TBI, TBI+ vehicle and TBI+quercetin (TBI+Que) (with three subgroups: 20, 50 and 100 mg/kg). The mice in the TBI+Que group were injected with quercetin (Sigma‐Aldrich, Shanghai, China) at 50 mg/kg intraperitoneally 30 min. after onset of TBI. At corresponding time‐points, all mice in the TBI+vehicle group were administered isometric of vehicle (50% DMSO). The mice from each group were killed 24 hrs post‐TBI.

### Brain tissue processing

For Western blot analysis, animals were killed 24 hrs post‐TBI, and ipsilateral cortex tissues were collected. The tissue was positioned directly over the centre of the injury site, covering both the contusion and the penumbra. The left cerebral cortex was removed and immersed in 4% paraformaldehyde overnight. For immunofluorescence staining, the whole brain was immersed in 4% paraformaldehyde overnight.

### Brain water content

The brain water content was measured in accordance with a previous study 20. After a neurological function test, mice were killed immediately. Subsequently, their brains were quickly removed and placed on a cooled brain matrix. The brain stem and the cerebellum were then removed. The fresh ipsilateral tissue was weighed to confirm the wet weight, dried for 72 hrs at 80°C and then weighed again to obtain the dry weight. The brain water content was calculated using the following formula: [(wet weight ‐ dry weight)/wet weight]×100%

### Isolation of mitochondria

Mitochondrial and cytosolic proteins were extracted from the left cerebral cortical tissue with the use of the Mitochondrial Isolation Kit for Tissue (Beyotime Institute of Biotechnology, Shanghai, China) 9. Fresh tissue samples were homogenized with a Polytron grinder in ice‐cold homogenization buffer and then centrifuged at 1000× *g* for 5 min. at 4°C to isolate the nuclear fraction. The obtained supernatants were centrifuged at 3500× *g* for 10 min. at 4°C to sediment the mitochondria. The supernatants were collected and then centrifuged at 12,000× *g* for 10 min. at 4°C to remove the sediment and obtain cytoplasmic proteins.

### Western blot analysis

Nuclear and cytoplasmic proteins were extracted in accordance with the instructions in the Nuclear and Cytoplasmic Protein Extraction Kit (Beyotime Biotech Inc, Nantong, China) 21. The nuclear proteins, mitochondrial proteins and total protein concentrations were then separated using 10% or 12% sodium dodecyl sulphate‐polyacrylamide gel and transferred electrophoretically to polyvinylidene difluoride membranes. The membranes were blocked for 2 hrs in 5% skimmed milk. The nuclear protein was incubated overnight at 4°C with PGC‐1α (1:1000 diluted, rabbit; Millipore, Billerica, MA, USA) and histone H3 (1:1000 diluted, rabbit; Cell Signaling Technology, Beverly, MA, USA) in blocking buffer. The cytoplasmic protein was incubated overnight at 4°C with PGC‐1α (1:1000 diluted, rabbit; Millipore), Bax (1:400 diluted, rabbit; Abcam, Cambridge, MA, USA), cytochrome c (1:1000 diluted, rabbit; Abcam), cleaved caspase‐3 (1:1000 diluted, rabbit; Cell Signaling Technology) and β‐actin (1:5000 diluted, rabbit; Bioworld Technology, St. Louis Park, MN, USA). and COX IV (1:1000 diluted, mouse; Cell Signaling Technology) in a diluent buffer. After three washes in Tris‐buffered saline with Triton X‐100 (TBST) for 10 min., the membranes were incubated with horseradish peroxidase (HRP)‐conjugated anti‐goat secondary antibodies (1:5000 diluted, goat or rabbit; Bioworld Technology) for 2 hrs at room temperature. After three washes with TBST for 15 min. each time, protein bands were visualized using enhanced chemiluminescence Western blot detection reagents (Millipore) and then detected on X‐ray film (Fuji Hyperfilm, Tokyo, Japan). Band density was quantified using Un‐Scan‐It 6.1 (Silk Scientific Inc, Orem, UT, USA); data were normalized to β‐actin, COX IV or histone H3.

### Immunohistochemistry

Consecutive coronal sections were cut at 4‐μm intervals to collect the lesioned cortex 22. After slides were routinely deparaffinized, endogenous peroxidase was quenched in 3% hydrogen peroxide and methanol. Non‐specific binding of antibodies was blocked with PBS containing 10% normal goat serum for 30 min. Primary anti‐PGC‐1α antibody (1:300 diluted, goat; Abcam) and caspase‐3 (1:300 diluted, goat; Cell Signaling Technology) were incubated overnight at 4°C. After three washes in PBS for 15 min. each, the sections were incubated with HRP‐conjugated IgG (1:500; Santa Cruz Biotechnology, Santa Cruz, CA, USA) for 60 min. at room temperature. After three washes with PBS, immunolabelling was visualized as brown by counterstaining with haematoxylin, dehydrated in ethanol and cleared in xylene. Six random vision fields (400×) in each coronary section were chosen as the data of each section. A total of four sections from each mouse were used for quantification. The final average number of the four sections was regarded as the data for each sample.

### Terminal deoxynucleotidyl transferase‐mediated dUTP nick‐end labeling (TUNEL) analysis

Formalin‐fixed tissues were embedded in paraffin and sectioned at 4 μm with the use of a microtome. The apoptotic cells were determined using a TUNEL detection kit (Roche, Indianapolis, IN, USA). The procedures were conducted in accordance with the instructions in the kit and as described in a previous study 23. Briefly, the sections were first deparaffinized and rehydrated and then washed with PBS, followed by digestion in proteinase K for 15 min. After washing with PBS twice for 5 min. each time (2 × 5 min.), the sections were incubated at 37°C with a labelling solution containing a TUNEL reaction fluid for 60 min. Subsequently, the sections were washed thrice with PBS for 10 min. each time (3 × 10 min.) and then blocked with 10% goat serum in 0.1 M Tris for 15 min. DNA was visualized by treating the tissue with a 1:40 dilution of streptavidin–HRP peroxidase and staining with DAB chromogen. The apoptotic cells exhibited cell shrinkage with condensed nuclei stained brown. For negative control purposes, several slides were incubated with a label solution not containing TdT. The distinctive morphological features of apoptosis were used to recognize apoptotic cells. The positive cells were identified, counted and analysed under a light microscope by an investigator blind to the grouping. The extent of brain damage was evaluated using the apoptotic index, defined as the average percentage of TUNEL‐positive cells in each section counted in 10 cortical microscopic fields (at 400× magnification). For the quantification of apoptotic cells, eight coronal sections of identical brain regions from the animals were selected (from approximately bregma ‐1.0 mm to bregma ‐3.0 mm at 200 μm intervals). The final average percentage of the TUNEL‐positive cells of the eight sections was regarded as the data for each sample.

### Mitochondrial superoxide dismutase (SOD) and malondialdehyde (MDA) content

Mitochondrial MDA content and SOD activity were measured using a spectrophotometer in accordance with the manufacturer's instructions (Nanjing Jiancheng, Nanjing, China). Total protein concentrations were determined using the Bradford method. The content of MDA was expressed as nmol/mg protein, whereas the activity of SOD was expressed as U/mg protein.

### Statistical analysis

SPSS 17.0 (SPSS Inc., Chicago, IL, USA) was used for the statistical analysis. Data were reported as mean ± S.E.M. Statistical analysis among groups was performed by one‐way anova, following Tukey's *post hoc* test. *P* < 0.05 was considered statistically significant.

## Results

### Quercetin alleviated cerebral oedema post‐TBI

**Figure 1:**
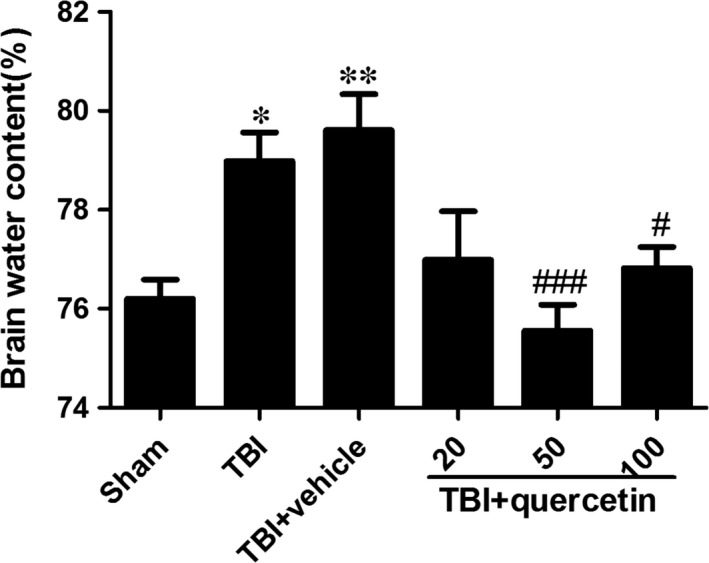
Brain water content was examined at 1 day after TBI. Brain water content was significant lower in the groups with administration of quercetin (20, 50, 100 mg/kg) than vehicle‐treated group. Data are presented as mean ± S.E.M., *P < 0.05, **P < 0.01 *versus* sham group; ^#^ *P* < 0.05, ^###^ *P* < 0.001 *versus* TBI + vehicle group.

The brain water content of the mice in the TBI groups was significantly greater than the sham group (Fig. 1). No significant difference in brain water content was indicated between the TBI group and the TBI+vehicle group (Fig. 1).

There was significantly less brain water in groups treated with quercetin than TBI+vehicle group (Fig. 1). However, larger doses such as 100 mg/kg failed to exhibit improved neuroprotection. Therefore, the results stated that quercetin has neuroprotective effect after TBI, and 50 mg/kg shows best result, which would be used in the following experiments.

### Quercetin decreased neuronal degeneration

**Figure 2:**
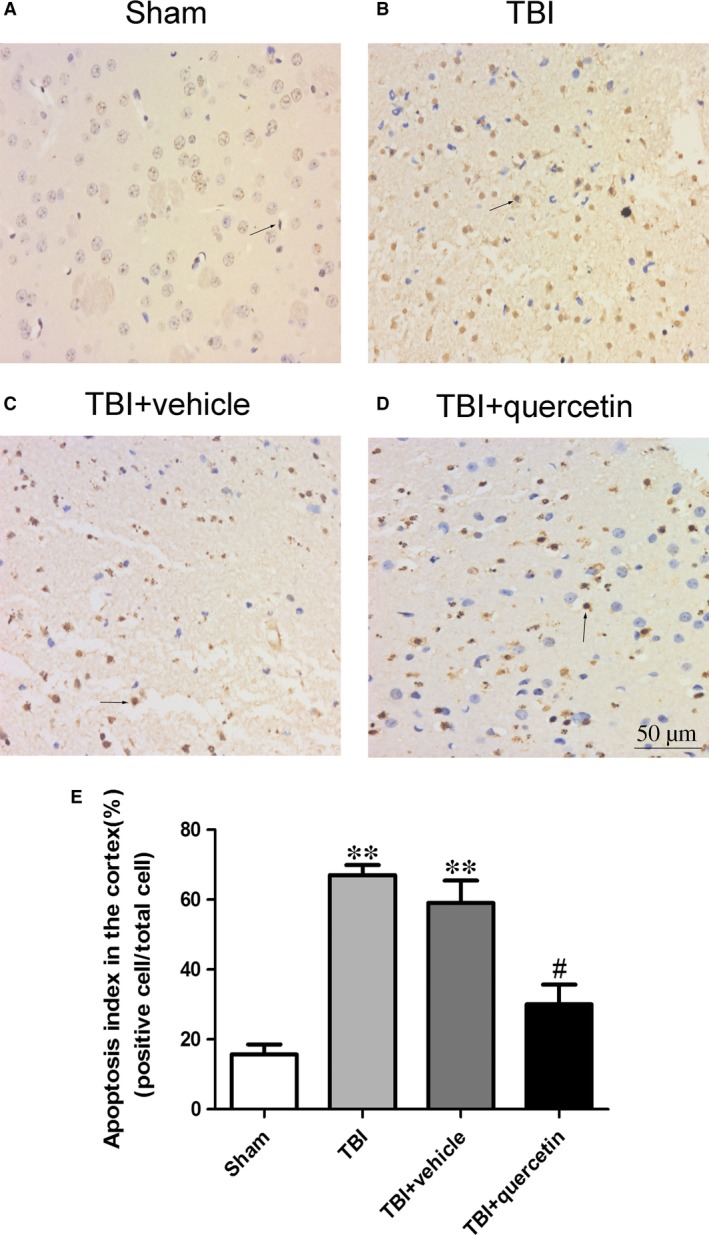
Apoptotic index was determined using TUNEL assays 1 day after TBI. Quercetin treatment significantly decreased the percentage of apoptotic cells after TBI(**A‐E**). Data are presented as mean ± S.E.M.; **P < 0.01 *versus* sham group; ^#^ *P* < 0.05 *versus* TBI + vehicle group.

As shown in Figure 2, few positive cells were found in the brain tissue of the sham group. Obviously, the apoptotic index was increased in the TBI+vehicle group relative to that in the sham group (Fig. 2A and C). No difference was indicated between the TBI group and the vehicle‐treated group; however, a decrease was found in the TBI+Que group (Fig. 2B–D). This result indicated that quercetin‐treated post‐TBI could result in fewer cell apoptosis surrounding the cortical contusion and could potentially ameliorate the secondary brain insult.

Quercetin promoted the translocation of PGC‐1α from the cytoplasm to the nucleus and enhanced PGC‐1α binding.

**Figure 3:**
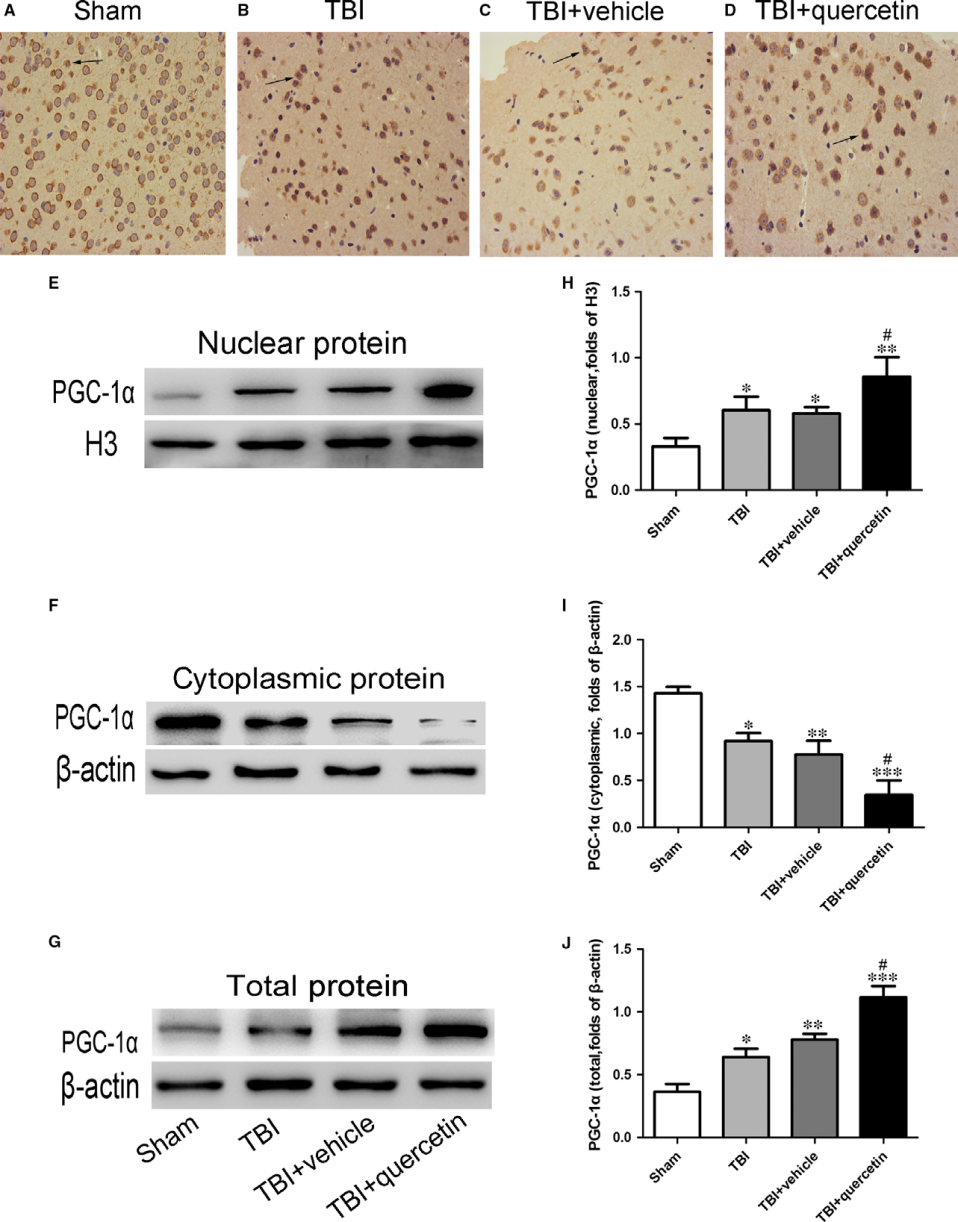
Quercetin promoted translocation of PGC‐1α from cytoplasm to nucleus and enhanced PGC‐1α binding. (**A‐D**) The representative photomicrographs showing PGC‐1α immunohistochemistry of tissue from different group 24 hrs after TBI. (**E, H**) The nuclear PGC‐1α expression after quercetin treatment in mice with TBI, as measured by Western blot. (**F, I**) The cytoplasmic protein PGC‐1α expression after quercetin treatment in mice with TBI, as measured by Western blot. (**G, J**) The total protein PGC‐1α expression after melatonin treatment in mice with TBI, as measured by Western blot. Bars represent the mean ± S.E.M. *P < 0.05, **P < 0.01 and ***P < 0.001 compared with the sham group; ^#^ *P* < 0.05 compared with the TBI + vehicle group. Black arrows: PGC‐1α‐positive neuron cell.

The ratios of cytoplasmic PGC‐1α, nuclear PGC‐1α and total PGC‐1α were investigated by Western blot analysis and immunohistochemistry. Compared with the sham group, both TBI and quercetin administration induced PGC‐1α nuclear translocation (Fig. 3A, B, D and E). Except that, compared with the TBI+vehicle group, the TBI+Que group exhibited significantly increased total PGC‐1α, which indicated that quercetin promoted the PGC‐1α level following TBI (Fig. 3G and H).

### Expression of caspase‐3 was down‐regulated by quercetin administration

**Figure 4:**
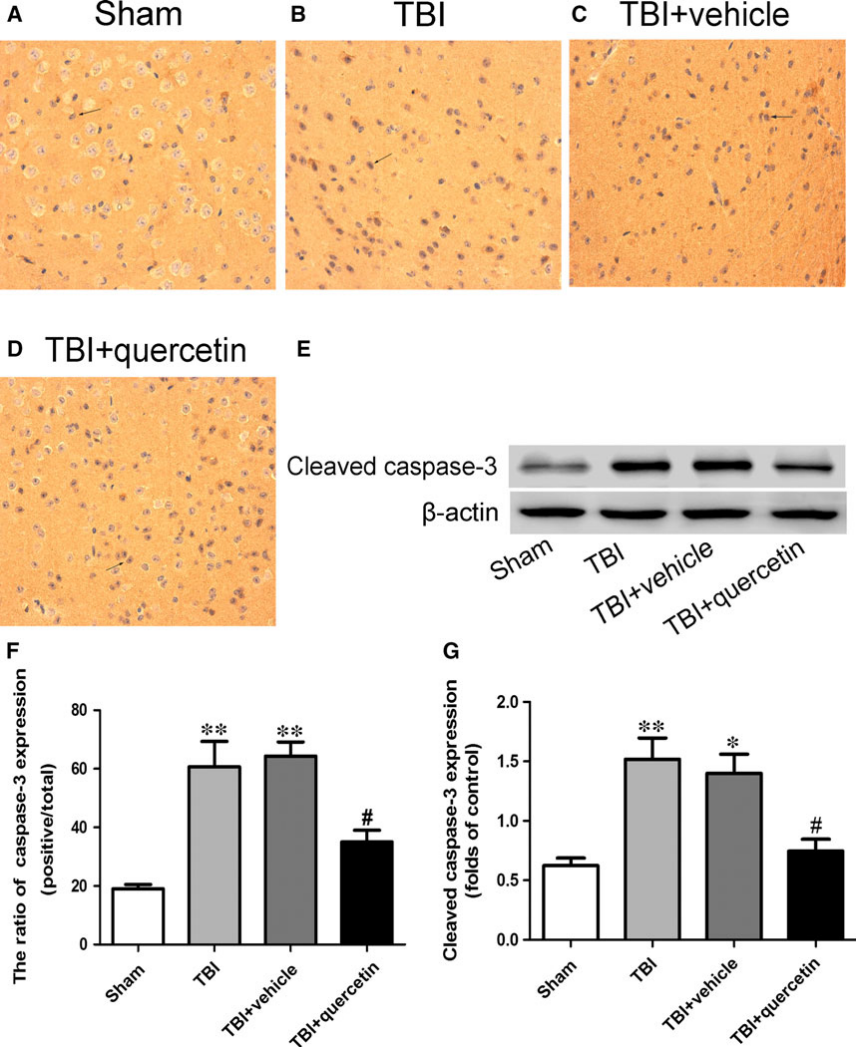
The representative photomicrographs showing caspase‐3 immunohistochemistry of tissue from different group 1 day after TBI (**A‐D, F**). Except that, effect of quercetin on cleaved caspase‐3 expression in cortical neural cells in a mice model of TBI was assessed by Western blot analysis (**E, G**). Data are presented as mean ± S.E.M. *P < 0.05, **P < 0.01 *versus*. sham group; ^#^ *P* < 0.05 *versus* TBI + vehicle group. Black arrows: caspase‐3‐positive neuron cell.

The protective effects of quercetin against neural apoptosis after TBI were detected by Western blot analysis and immunohistochemistry of mouse brain tissues. The number of caspase‐3‐positive cells in the quercetin administration group was fewer than the vehicle treatment, and the cleaved caspase‐3 expression rose 24 hrs following TBI (Fig. 4A–E and G).

**Figure 5:**
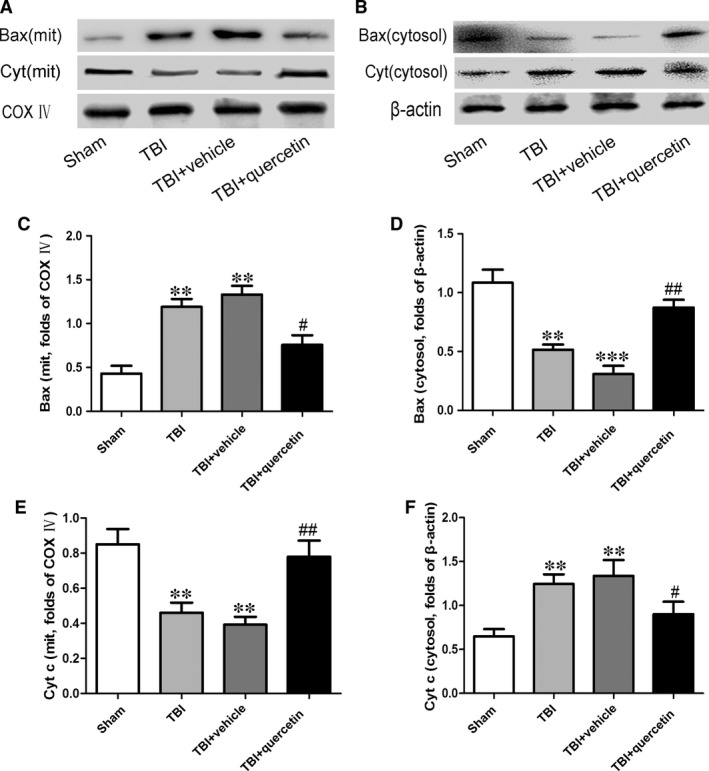
Effect of quercetin on pro‐apoptotic protein expression was assessed following TBI. (**A, B**) The expression of Bax and cytochrome c in the ipsilateral cortex was evaluated by Western blotting 24 hrs after injury. Representative blots show the relative expression of (**C, E**) mitochondrial and (**D, F**) cytosolic Bax and cytochrome c. Expression was normalized to the level of COX IV or β‐actin. Data represent the mean ± S.E.M. (*n* = 6 per group). **P < 0.01, ***P < 0.001 *versus* sham group; ^#^ *P* < 0.05, ^##^ *P* < 0.05 *versus* TBI.

Compared with the sham group, the levels of Bax protein in the mitochondria were increased, whereas those in the cytosol were decreased significantly (Fig. 5A–D). The mitochondrial cytochrome c levels were decreased, whereas the cytosolic cytochrome c levels were increased (Fig. 5A, B, E and F).

### Quercetin reduces oxidative stress in the mitochondria of injured brain

**Figure 6:**
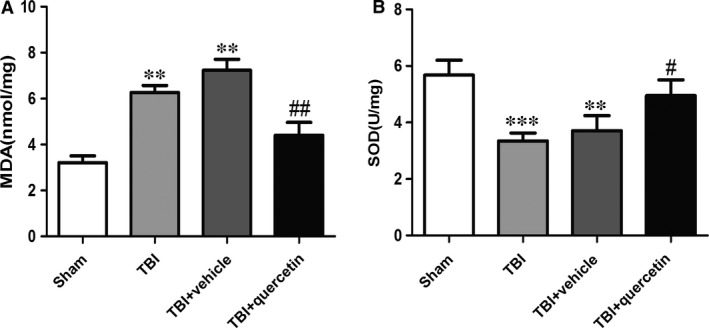
Quercetin attenuated mitochondrial oxidative stress caused by TBI. (**A**) Measurements of MDA levels (*n* = 6 per group). (**B**) The activities of SOD. Data represent the mean ± S.E.M. **P < 0.01, ***P < 0.001 *versus* sham group; ^#^ *P* < 0.05, ^##^ *P* < 0.01 *versus* TBI+vehicle.

Mitochondrial MDA was investigated in the TBI, TBI+vehicle and TBI+Que groups (Fig. 6A). Administration of quercetin reduced the generation of mitochondrial MDA. By contrast, mitochondrial SOD was decreased after TBI. Quercetin significantly up‐regulated the activity of SOD (Fig. 6B).

## Discussion

A number of studies have indicated mitochondrial participation in traumatic neuronal injury 24, 25. Thus, neuroprotective approaches must include strategies aimed at limiting and reversing mitochondrial dysfunction. However, previous studies indicated that quercetin can prompt mitochondrial electron transportation while stimulating chain anti‐ROS reaction for limiting the damage connected with defective mitochondrial function in many neuron diseases 26, 27. Studies on the internal mechanisms of this appearance have rarely been conducted, and no evidence of the effect of quercetin has been indicated in this process following TBI. Therefore, the effects of quercetin on mitochondrial function adaptation and the PGC‐1α protein levels in the mouse brain were examined. In this study, treatment with quercetin (50 mg/kg) attenuated contusion‐induced brain oedema and alleviated neuronal apoptosis 12, 28. And else, quercetin administration was shown to stimulate mitochondrial biogenesis by the activation of the PGC‐1α pathway. The present study is the first, thus far, to estimate the effects of quercetin in modulating the mitochondrial activity *via* the PGC‐1α signalling pathway in the mouse model of TBI.

Mitochondria plays very important role in the injured brain 29, 30, 31, 32. Bax belongs to the Bcl‐2 family proteins which is a downstream factor in the mitochondrial apoptosis pathway 33. Mitochondrial Bax forms a channel permeable to cytochrome c, which only exists in the mitochondrial intermembrane space 34, 35, 36. Cytochrome c released from the mitochondria results in the sequential activation of caspases‐3 37. The present study showed that the release of cytochrome c into the cytosol and the translocation of Bax to the mitochondrial membrane were increased significantly after TBI. The results indicated that the mitochondria lesion in brain tissue was activated after TBI. However, all of these changes led to the levels of cleaved caspase‐3 up‐regulation, which leads to neuronal apoptosis. MDA has been considered the index of lipid peroxidation. In addition, SOD is an antioxidant enzyme, involved in the catalytic detoxification of superoxide 38, 39. The conversion content of MDA and the activity of SOD in the TBI cortex of mouse brain mitochondria proved that oxidative stress occurred following TBI. These results suggest that quercetin protects neurons by restraining the mitochondrial apoptosis pathway.

PGC‐1α is a multifunctional protein which activates many nuclear receptors and functions. Actually, PGC‐1α plays an important position in many nervous system diseases 2. It was demonstrated that the nuclear PGC‐1α protein level was significantly increased after TBI 18. To confirm the involvement of the PGC‐1α signalling pathway in the protective role of the injured cortex of mouse brain mitochondria, the changes in the PGC‐1α signalling pathway were investigated after quercetin administration 1, 2, 3, 4. The data in the current study confirmed that PGC‐1α transferred from the cytoplasm to the nucleus following TBI and that quercetin facilitated this process. The nuclear PGC‐1α protein was up‐regulated significantly post‐TBI and was considerably higher after quercetin administration. The total protein of PGC‐1α also increased significantly when quercetin was supplied. These results implied that quercetin not only promoted PGC‐1α translocation from the cytoplasm to the nucleus but also urged protein expression in the experimental model of TBI.

In summary, this study confirms that PGC‐1α plays an important role in mitochondrial biogenesis after TBI. Likewise, this study suggests that quercetin treatment in regions of the TBI mouse brain directly induces the level of PGC‐1α expression up‐regulation. The increase in PGC‐1α levels suggested that PGC‐1α may detect an alteration in energy demand, such as the change of ATP levels. Regardless, a further investigation has to be conducted on the underlying mechanism of PGC‐1α translocation.

## Conflict of interest

None.
